# Ultrasound-responsive phase-transitional nanomedicine enables intensity-tunable postoperative analgesia

**DOI:** 10.3389/fbioe.2025.1704679

**Published:** 2025-10-27

**Authors:** Xinye Song, Miao Feng, Hao Chen, Yong Luan

**Affiliations:** ^1^ Department of Anesthesiology, The First Affiliated Hospital of Dalian Medical University, Dalian, Liaoning, China; ^2^ Department of Gastroenterology, The First Affiliated Hospital of Dalian Medical University, Dalian, Liaoning, China

**Keywords:** ultrasound-triggered release, phase transition, postoperative analgesia, theranostics, nanoplatform

## Abstract

**Introduction:**

Effective handling of pain after surgery is a major clinical issue, since insufficient pain relief is associated with extended recovery, excessive opioid use, and increased healthcare. Current approaches are limited by the short duration of local anesthetics, opioid-related adverse effects, and the lack of dynamic adjustability in pain relief. Here we report a theranostic nanoplatform, Rg3-liposomes@DMSN-levobupivacaine-PFP (RDLP), which integrates ultrasound-triggered phase transition, contrast-enhanced ultrasound (CEUS) imaging, and intensity-tunable drug release to address these limitations.

**Methods:**

RDLP features a core-shell architecture: dendritic mesoporous silica nanoparticles (DMSN) encapsulate the local anesthetic levobupivacaine and the phase-transition agent perfluoropentane (PFP), with a biocompatible Rg3-liposome coating enhancing stability and reducing drug leakage. Upon ultrasound irradiation, PFP undergoes liquid-to-gas phase transition, generating microbubbles that amplify CEUS signals for real-time visualization of drug distribution and drive inertial cavitation to trigger burst release of levobupivacaine. This design achieves high levobupivacaine encapsulation efficiency and enables spatiotemporally controlled release, with ultrasound accelerating drug release kinetics *in vitro*.

**Results:**

RDLP combined with ultrasound prolonged analgesia compared to free levobupivacaine and enabled on-demand adjustment of pain relief intensity via multiple ultrasound irradiation cycles, restoring paw withdrawal thresholds and latencies to near-baseline levels *in vivo*. The platform exhibits exceptional biocompatibility, with no histopathological damage to sciatic nerves.

**Discussion:**

RDLP bridges imaging guidance and therapeutic intervention, leveraging ultrasound’s deep tissue penetration and Rg3’s natural biocompatibility to overcome limitations of conventional nerve blocks and ultrasound-responsive systems. This non-invasive, adjustable strategy offering potential to reduce opioid reliance and improve patient outcomes in perioperative care.

## 1 Introduction

Postoperative pain constitutes an unavoidable issue tied to prolonged recovery periods, heightened opioid consumption, and elevated healthcare expenses when inadequately managed (Kehlet et al., 2006; Slomski, 2022). As a common perioperative complication, it affects roughly half of all patients within 24 h after surgery, varying with clinical surgical types. Moderate pain occurs in approximately 40% of cases, whereas severe pain ranges from 10% to 50% (Kehlet, 2018). Moreover, a significant portion of acute pain transitions into chronic pain. Consequently, effective postoperative analgesia offers substantial economic advantages by enhancing patient quality of life, aligning with enhanced recovery after surgery principles (Qiao et al., 2023; Qiao et al., 2022). Despite employing multiple analgesics, only about 50% of patients achieve adequate pain relief, partly influenced by the specific surgery and analgesic or anesthetic techniques applied (Admiraal et al., 2021).

Local anesthesia offers postoperative pain control while avoiding opioid-related risks (Pascarella et al., 2021; Hazam et al., 2024). However, the limited duration of local anesthetics, typically 2–4 h, restricts their analgesic use. Levobupivacaine is a longer-acting option with higher therapeutic index, enhanced sensory block selectivity, reduced systemic toxicity, making it better suited for nerve blocks (Dubey et al., 2024). Achieving prolonged analgesia requires repeated invasive injections, risking local tissue damage or neurotoxicity from drug accumulation. Responsive drug delivery systems offer dynamic pain control through adjustable drug release profiles while minimizing opioid dependence (Liang et al., 2022; Luo et al., 2020; Yin et al., 2022). These advanced systems permit precise modulation of analgesic parameters including duration, anatomical targeting, and dosage intensity via external activation mechanisms. Photonic stimulation has gained attention as a potential triggering modality due to its adjustable wavelength, irradiance zone, and temporal control (Cheng et al., 2021; Qiao et al., 2020). A critical limitation persists in the limited tissue penetration capacity of visible light wavelengths, presenting a significant barrier for clinical implementation of light-activated anesthetic delivery systems (Jiang et al., 2024; Zheng et al., 2023; Liao et al., 2023). In clinical settings, sonography has become a crucial modality for targeted pain management due to its enhanced tissue penetration capabilities (Luo et al., 2022; Xin et al., 2024). This imaging technology enables precise visualization of neural pathways, osseous structures, and adjacent vasculature during analgesic procedures. When contrasted with conventional landmark-based nerve block techniques dependent on operator expertise, ultrasonography-guided approaches demonstrate improved procedural accuracy while minimizing iatrogenic risks. The development of real-time imaging-assisted, dosage-modulated analgesia systems therefore represents a vital advancement goal in contemporary pain management research.

Recent advancements in ultrasound-based diagnostic techniques and analgesic therapies have progressed in tandem (Kim et al., 2023; Sabetian, 2025; Kim et al., 2021). The clinical use of ultrasound confirms its biological safety and improves the efficiency of anti-tumor drug delivery (Xie et al., 2023; Liu et al., 2020). Ultrasound-driven microbubbles can enhance medication transport through inert cavitation and increased interstitial fluid flow. However, microbubbles exhibit instability in physiological conditions, and their short half-life limits sustained pain relief (Fournier et al., 2023). Liquid-to-gas phase-transitional nanomedicine demonstrates enhanced structural integrity for diagnostic imaging applications in theranostics (Jiang et al., 2022). Acoustic energy induction initiates liquid perfluoropentane (PFP) phase transformation, generating gas microbubbles for enhanced visualization (Zhong et al., 2019). These responsive contrast media simultaneously modify the acoustic microenvironment to amplify pharmaceutical transport efficiency through inert cavitation.

Ginsenoside has recently sparked considerable activity in nanotechnology owing to its distinct physical and chemical attributes (Xi et al., 2022). Rg3, a classical ginsenoside with excellent biocompatibility, holds strong promise for drug delivery due to its innate ease of fabrication (Fan et al., 2024). Our study presents an experimental model for postoperative analgesia that synchronizes controlled pharmacological release with physiological feedback mechanisms. The application of liquid-to-gas phase transition technology enabled substantial reduction in acoustic energy thresholds. Contrasting with inert nanoparticles lacking acoustic responsiveness, this methodology achieves comparable cavitation while reducing ultrasonic energy exposure and minimizing adverse effects. Growing evidence highlights the importance of external stimuli in modulating pain intensity, prompting our focus on ultrasound-triggered, adjustable pain management. Leveraging these insights, we designed a drug delivery system using dendritic mesoporous silica nanoparticles (DMSN), incorporating phase-transitional perfluoropentane (PFP) and the low-molecular-weight anesthetic levobupivacaine, and coated it with Rg3-based liposomes, termed Rg3-liposomes@DMSN-levobupivacaine-PFP (RDLP). Within this nanoplatform, RDLP induces a liquid-to-gas shift, generating a gas environment that enhances ultrasound-triggered levobupivacaine release under moderate-intensity sonication and provides imaging signals for contrast-enhanced ultrasonography. The therapeutic combination of ultrasound exposure and RDLP facilitates precision-controlled analgesia through ultrasound-triggered drug delivery, with real-time monitoring via ultrasound imaging. Our findings show that RDLP combined with ultrasound irradiation markedly reduces local pain with tunable intensity. This study underscores the potential of an ultrasound-guided strategy for intensity-adjustable pain management.

## 2 Materials and methods

### 2.1 Materials

Levobupivacaine hydrochloride was sourced from Aladdin Biochemical Technology Co., Ltd. in Shanghai, China. PL-100M nanoparticles were acquired from AVT Pharmaceutical Technology Co., Ltd. based in Suzhou, China. DAPI fluorescent dye was obtained through Beyotime Biotechnology Institute located in Shanghai, China. The standard CCK-8 cell proliferation assay kit was purchased from Dojindo Molecular Technologies, Inc. in Kumamoto, Japan. PFP was supplied by J&K Science Ltd. Shanghai Yuanye Biotechnology provided dialysis membranes with 3,500 Da molecular weight cutoff. CTAC, TEOS, TEA, and BTES were obtained from Sigma-Aldrich Co., LLC. All chemical substances maintained analytical or reagent-grade purity and were used without further purification.

### 2.2 Fabrication of DMSN

DMSN was fabricated using an established methodology based on a sol-gel process. Initially, CTAC (2 g) and TEA were dissolved in deionized water (20 mL) under continuous mechanical agitation at a temperature of 95 °C. After a 20-min equilibration period to ensure homogeneity, BTES (1.3 g) and TEOS (1.0 g) were gradually introduced into the mixture through precise dropwise addition over a duration of 4 h, maintaining constant stirring to facilitate controlled hydrolysis and condensation reactions. The reaction mixture was then cooled to room temperature, allowing the synthesized particles to precipitate. These particles underwent multiple sequential washes with absolute ethanol and deionized water, each wash involving centrifugation and decantation to effectively remove residual reactants and byproducts. To extract the CTAC surfactant template, the purified samples underwent reflux treatment at 78 °C for 12 h in an ethanol-based hydrochloric acid solution (10% v/v HCl). Following the reflux, the samples were centrifuged, rinsed thoroughly with ethanol to neutralize acidity, and dried under vacuum at 60 °C to obtain the final DMSN product for further use.

### 2.3 Drug loading procedure for DMSN

An aqueous dispersion of DMSN (10 mg in 5 mL of pure water) was mixed with levobupivacaine (10 mg) and kept stirring under ambient conditions overnight. Subsequently, PFP (200 μL) was incorporated into the mixture with ice-cooled agitation for an additional 12-h period. The final DMSN-levobupivacaine-PFP (DLP) composite was isolated through repeated centrifugations.

### 2.4 Preparation of liposomes and RDLP nanoparticles

Rg3-liposomes were fabricated via the thin-film hydration method. A blend of PL-100M and Rg3 in a 10:3 weight ratio was dissolved in a chloroform-ethanol solvent system (1:1 volume ratio). This mixture underwent solvent evaporation under reduced pressure at 48 °C for 2 h to form a uniform lipid film. This film was hydrated with a 5% w/v glucose solution at 48 °C for 1 h. The resulting suspension was probe-sonicated (300 W, 20 kHz, 2 min) to yield Rg3-liposomes.

RDLP nanoparticles were fabricated via extrusion-induced liposome coating. The preparation process involved suspending 0.9 mg of DLP nanoparticles in 5 mL of ultrapure water and gradually mixing with 0.9 mg of Rg3-liposomes through dropwise addition while maintaining constant agitation at 500 rpm. This mixture underwent multiple cycles of extrusion through a polycarbonate membrane to promote liposomal fusion onto nanoparticle surfaces. Post-extrusion purification involved subjecting the suspension to centrifugation under refrigeration (4 °C), followed by triple rinsing with cold ultrapure water to eliminate non-adsorbed liposomes. The resulting supernatant was subsequently analyzed by HPLC to quantify unencapsulated levobupivacaine.

### 2.5 Levobupivacaine release pattern *in vitro* from DLP and RDLP nanoparticles

DLP and RDLP nanoparticles were encapsulated within dialysis membranes (5 mg mL^-1^, 3.5 kDa cutoff) and immersed in 20 mL of deionized water. The system underwent continuous agitation at 160 rpms on a thermostatic orbital shaker (JINGHONG). To investigate ltrasound-triggered drug release, Biofil tubes containing the nanoparticles received six cycles of non-focused ultrasound treatment from SXUltrasonic’s 90 mm planar transducer, using acoustic coupling gel for optimal energy transfer. Ultrasound parameters included 0.6 W cm^-2^ intensity, 1.0 MHz frequency, 15 min duration, and 50% duty cycle. Control samples omitted ultrasound. At set intervals, 5 mL of release medium was sampled to quantify levobupivacaine. Cumulative release was assessed using UV-visible spectroscopy absorbance and a standard calibration curve.

### 2.6 Cytotoxicity assessment

Cultured DRG cells were plated in 96-well plates and allowed to adhere overnight. Following medium removal, cellular specimens were exposed to varying RDLP concentrations in RPMI-1640 medium, maintained under incubation for either 12 or 24 h. The culture medium was substituted with CCK-8 solution for 1–2 h of additional incubation. For ultrasound safety evaluation, cellular samples were treated with RDLP concentrations ranging from 0 to 600 μg mL^-1^ (0, 50, 100, 200, 300, 400, 500, and 600 μg mL^-1^) for 4 h. Post triple-rinsing with PBS to eliminate nanoparticle residues, specimens were exposed to ultrasound irradiation (0.6 W cm^-2^, 1.0 MHz, 50% duty cycle, 2 min duration). Culture medium containing 10% CCK-8 solution was subsequently introduced for viability assessment.

### 2.7 Detection of liquid-gas phase transition dynamics

The phase transition characteristics of RDLP nanoparticles were investigated under controlled ultrasound parameters (1.0 MHz, 0.6 W cm^-2^, 50% duty cycle). Ultrasonic imaging analysis (Canon i900, 4–15 MHz probe) was performed on RDLP nanoparticles. Continuous ultrasonic exposure was appliedand ultrasonic images with CEUS-Mode were collected pre- and post-irradiation. The echo intensity of the ultrasonic images were analyzed with the Image-Pro Plus 6.0 software. Light microscopy images were captured using a Nikon DS-Ri2 to document the RDLP samples.

### 2.8 Animal model and ethics statement

Male Balb/c mice, aged 4–6 weeks, while provided sufficient access to food and water. All experimental protocols involving animals were conducted in compliance with institutional guidelines for humane treatment and laboratory animal welfare. The study design received formal approval from the institutional ethics committee prior to commencement. All experimental protocols received ethical approval from Dalian Medical University’s Institutional Animal Care and Use Committee and strictly adhered to relevant regulatory standards.

### 2.9 Surgical protocol for mice incisional pain modeling

The experimental pain model was developed through standardized surgical techniques (Starkl et al., 2024). After administering brief anesthesia using 2% isoflurane for inhalation, the operative site underwent antisepsis. A small longitudinal incision was created extending from the proximal heel margin, followed by complete myotomy through elevation and full-length sectioning of the underlying muscle tissue. The incision was subsequently closed with suture.

### 2.10 RDLP injection and ultrasound treatment

The experimental animals received a single local administration of RDLP suspension, RDL formulation, levobupivacaine solution, or phosphate-buffered saline (containing 50 μg levobupivacaine in 100 μL volume, n = 5 per group) near the sciatic nerve region. Subsequently, subjects were restrained and their hind limb underwent ultrasound irradiation using a therapeutic system (1.0 MHz frequency, 0.6 W/cm^2^ intensity, 2-min duration, 50% duty cycle) at specified intervals-specifically at 48 h and 60 h post-administration for RDL + US and RDLP + US cohorts, while PBS + US group received treatments at predetermined time points.

### 2.11 *In vivo* monitoring with ultrasound

To track phase transition and distribution *in vivo*, we performed CEUS imaging after RDLP administration. Mice underwent ultrasound scanning using an Aplio i900 system equipped with an 18 MHz probe, with scans conducted at 15-min intervals. Each mouse received a standardized 0.05 mg levobupivacaine dose (formulated in 100 μL RDLP solution) administered through perioperative site infiltration. Quantitative analysis of image figures determined mean gray values.

### 2.12 Assessment of mechanical allodynia

Mechanical allodynia assessment was performed to quantify sensitivity to innocuous mechanical stimuli utilizing the KW-CT system (Kaerwen Inc., China) (Mitsui et al., 2023). Following equipment preparation, animals were acclimated on a silk mesh platform housed in clear chambers for environmental habituation (≥30 min). Gradual pressure application to the hindpaw was implemented through a calibrated probe, with the minimal force triggering paw withdrawal documented as the mechanical threshold. Five consecutive trials were conducted per subject, with mean paw withdrawal threshold (PWT) values recorded.

### 2.13 Assessment of thermodynamic hyperalgesia

Thermal nociception was evaluated using a heated surface apparatus (55 °C) (Guo et al., 2025). Paw withdrawal latency (PWL) was recorded upon withdrawal of the affected hind limb, with three consecutive trials performed per subject. Testing intervals maintained 5–10 min rest periods between measurements, incorporating a 20-s safety cutoff to prevent tissue damage. Triplicate measurements were averaged for subsequent data interpretation.

### 2.14 Statistical analysis

Statistical analyses were conducted utilizing GraphPad Prism 9 software. Experimental results are expressed as mean values accompanied by standard deviations (SD), with comparative assessments performed through Student's t-test and one-way ANOVA.

## 3 Results and discussion

### 3.1 Characterization of RDLP

**FIGURE 1:**
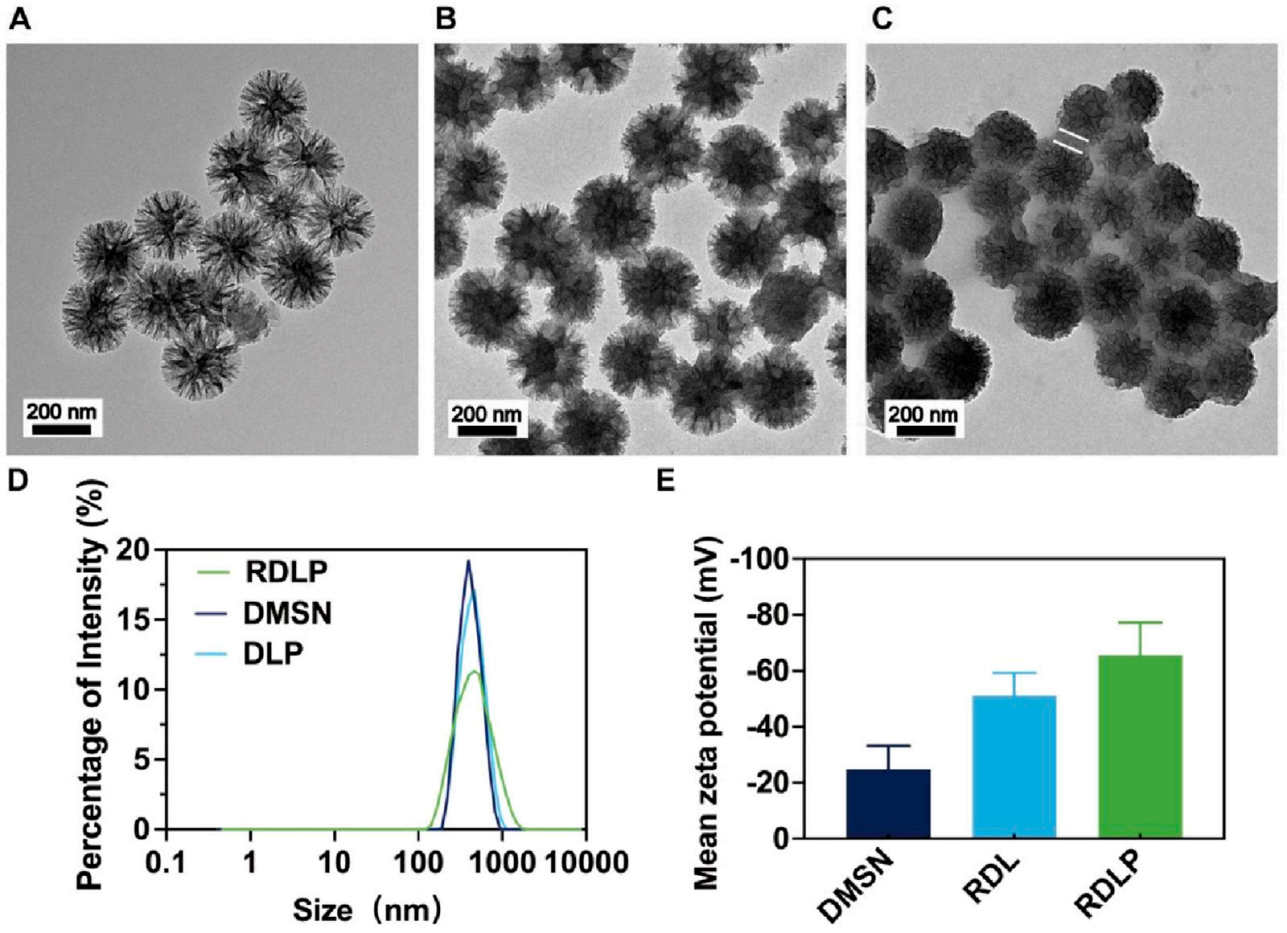
**(A)** TEM image of DMSN. **(B)** TEM image of DMSN-levobupivacaine-PFP (DLP). **(C)** TEM image of RDLP. The arrows in the figure shows the liposomes. **(D)** Size distribution of different nanoparticles. **(E)** Mean zeta potentials of different nanoparticles (n = 3).

DMSN functioned as the encapsulation matrix for PFP and levobupivacaine hydrochloride, homogenized in PBS through continuous agitation within a chilled environment. Transmission electron microscopy (TEM) characterization demonstrated that the synthesized DMSN exhibited consistent dendritic architecture (Figure 1A). Post-loading, TEM images indicated filling of the internal structure, evidenced by increased particle opacity (Figure 1B). Rg3-based liposome-camouflaged DLP nanoparticles (RDLP) were fabricated by extruding a mixture of Rg3-derived liposomes and DLP nanoparticles 11 times using a mini-extruder. TEM characterization confirmed the RDLP nanoparticles adopted a spherical core-shell structure following Rg3 liposome coating (Figure 1C). Dynamic light scattering (DLS) measurements showed a consistent increase in the hydrodynamic diameter of RDLP(PDI:0.317) compared to uncoated DLP nanoparticles (PDI:0.236) (Figure 1D). The zeta potential shifted from approximately −24.80 mV for DMSN to around −65.57 mV for RDLP (Figure 1E). The isotherm hysteresis loop was characteristic of mesoporous materials. DMSN exhibited a high Brunauer-Emmett-Teller (BET) specific surface area of 480 m^2^ g^-1^ and an average pore diameter of 13 nm, which indicates its substantial porosity. The encapsulation efficiency of levobupivacaine was determined as 91.3% by UV-Vis-NIR absorption spectroscopy. These results confirm the effective integration of DLP nanoparticles and Rg3 liposomes.

### 3.2 Ultrasound-responsive imaging and levobupivacaine release

**FIGURE 2:**
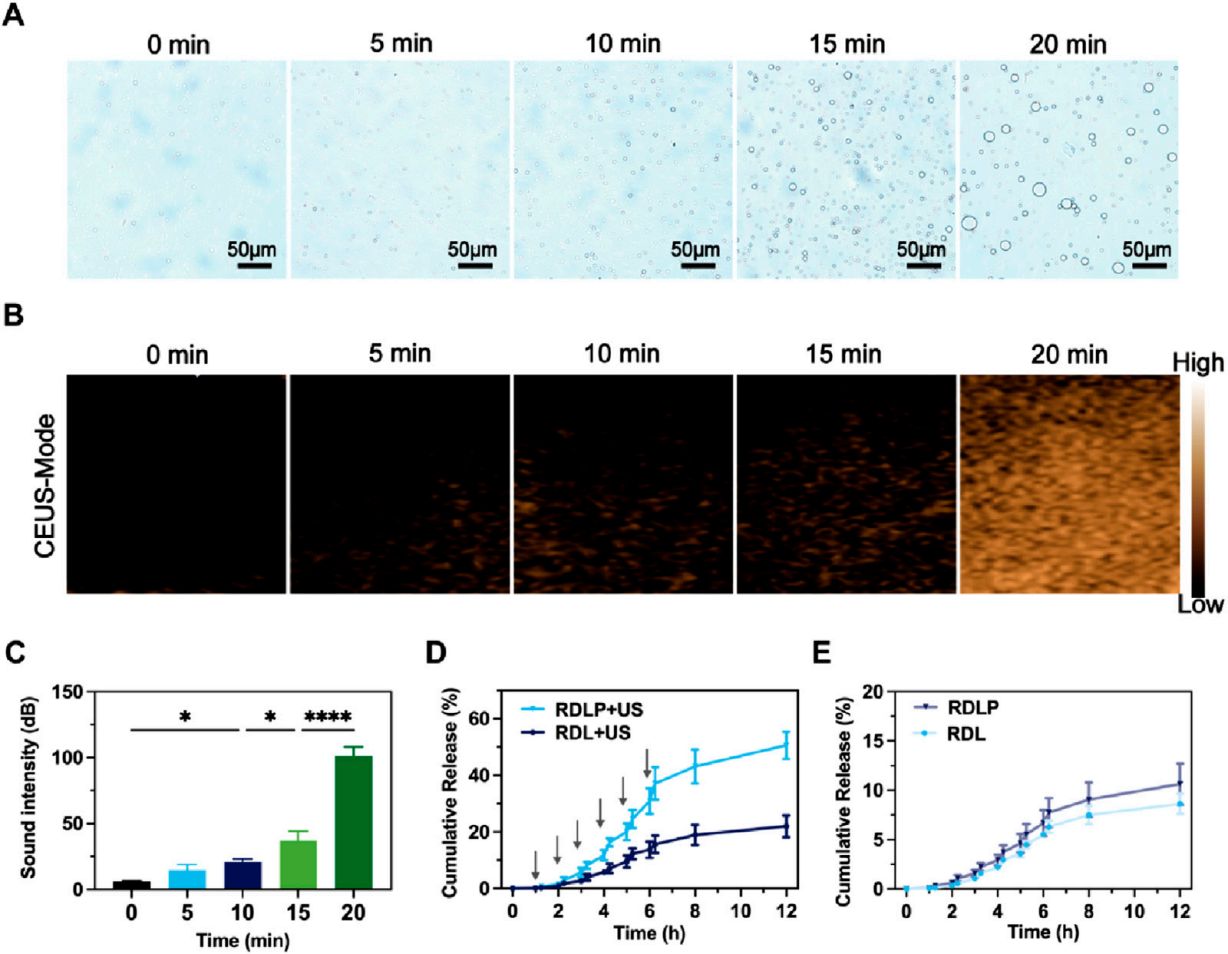
**(A)** Optical microscope images of RDLP post-ultrasound exposure at varying intervals. **(B)** CEUS images of RDLP after ultrasound treatment across different durations, and **(C)** associated quantitative sound intensity measurements. Cumulative release profiles of levobupivacaine in RDL and RDLP **(D)** under ultrasound influence or **(E)** in its absence (n = 3).

Following phase transformation, PFP-encapsulated nanomedicine demonstrated enhanced suitability for CEUS imaging and elevated cavitation performance (Yang et al., 2020). Consequently, we monitored changes in RDLP nanoparticles under ultrasound at specific intervals using light microscopy. Unlike NIR-responsive phase transitions involving PFP, ultrasound’s significant penetration depth offers greater clinical promise for this process (Gao et al., 2024). The dramatic expansion of RDLP from nano- to micro-scale enables CEUS imaging (Figure 2A). Experimental verification confirmed that ultrasound-activated PFP-loaded RDLP nanoparticles generated amplified CEUS signals through gas-phase expansion mechanisms (Figure 2B). Notably, quantitative analysis revealed a signal intensification post 20-min sonication (Figure 2C), attributable to microbubble generation. These findings confirm RDLP nanoparticle utility in CEUS imaging.

We assessed levobupivacaine release patterns following ultrasound exposure (1.0 MHz, 0.6 W cm^-2^, 50% duty cycle). The application of ultrasound can be synchronized with the onset and location of a patient’s pain, allowing precise spatiotemporal regulation of analgesic delivery. Furthermore, differing levobupivacaine release profiles between RDLP and RDL highlight this temporal control (Figure 2D). RDLP facilitated faster levobupivacaine release under identical ultrasound conditions, demonstrating ultrasound responsiveness. This suggests PFP-mediated liquid-to-gas transition and cavitation effectively boost drug release. Control experiments showed both RDLP and RDL released levobupivacaine slowly without ultrasound (Figure 2E). This property underscores the critical role of ultrasound-responsive RDLP nanostructures for temporally regulated pain intervention strategies.

### 3.3 Biosafety of RDLP treated with ultrasound

**FIGURE 3:**
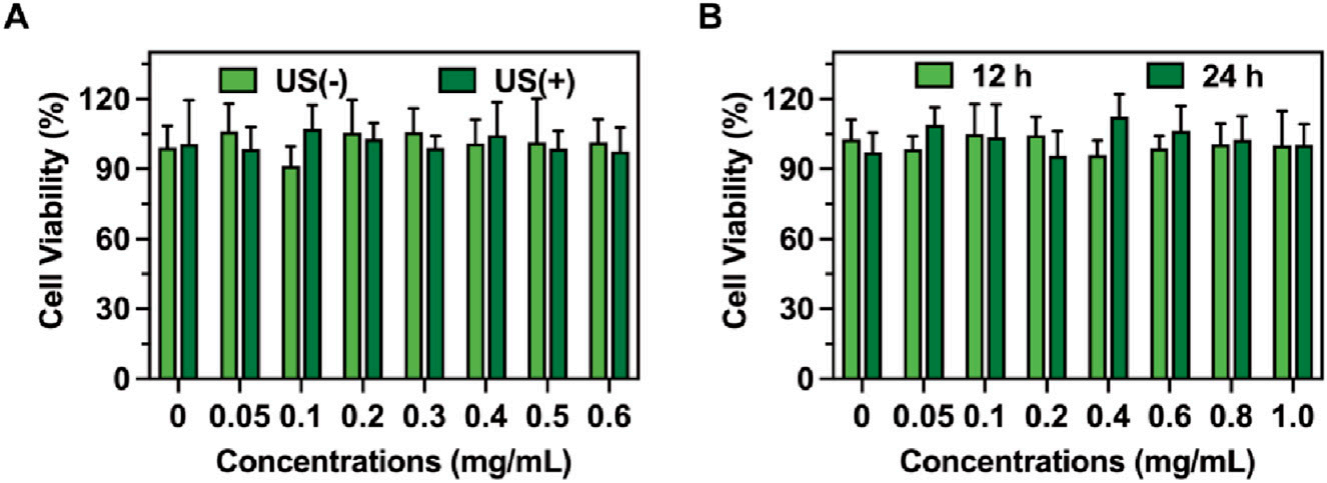
**(A)** Cell viability of RDLP cells after co-culturing with DRG cells with or without ultrasound. **(B)** Cell viability of RDLP following incubation with DRG cells for 12 h and 24 h (n = 5).

The biocompatibility of RDLP combined with ultrasound exposure was validated through standardized CCK-8 assays. Following a 4-h incubation period with RDLP, the culture medium was refreshed prior to administering ultrasound treatment to the cellular samples. As illustrated in Figure 3A, cell viability exhibited no significant differences between groups. These findings suggest that the analgesic protocol minimally influenced cellular proliferation patterns, confirming the favorable safety characteristics of our demand-responsive pain regulation approach. Furthermore, RDLP maintained high compatibility after 12 h and 24 h of incubation (Figure 3B).

### 3.4 Ultrasound imaging of RDLP *in vivo*

**FIGURE 4:**
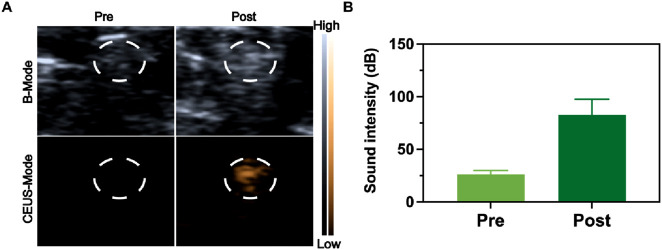
**(A)** Ultrasound visualization of the sciatic nerve following RDLP injection, captured in both B-mode and CEUS-mode. The circled area in the images identifies the sciatic nerve zone. **(B)** Intensities assessment of CEUS images restricted to the encircled region (n = 3).

Ultrasound-guided nerve blockade serves as an effective clinical approach due to ultrasound’s high convenience as an imaging tool for visualizing nerves, soft tissues, and vascular structures. Consequently, ultrasound-based drug release monitoring facilitates the evaluation of nerve blocks *in vivo*. Microbubbles driven by ultrasound can boost medication transport via inert cavitation and augmented interstitial fluid flow. Nevertheless, microbubbles show instability under physiological conditions, and their brief half-life restricts sustained pain alleviation. Nanomedicine with liquid -to-gas phase transition presents improved structural integrity for diagnostic imaging uses in theranostics. In our research, *in vivo* vaporization outcomes were assessed by tracking the CEUS signal, as PFP within RDLP nanoparticles acts as a multifunctional probe for visualizing RDLP nanoparticle distribution in pain management. Distinct CEUS enhancement in the combined RDLP + ultrasound cohort emerged following 15-min sonication (Figure 4A), while control group showed no significant contrast variation, confirming ultrasound-activated phase transition of nanocarriers in biological systems. Comparative analysis of contrast patterns between experimental and control groups demonstrated that ultrasound irradiation demonstrated exceptional imaging contrast capabilities for monitoring the phase-transitional phenomenon (Figure 4B), proving particularly valuable for real-time ultrasound imaging of RDLP nanoparticle behavior.

### 3.5 *In vivo* pain management using ultrasound

**FIGURE 5:**
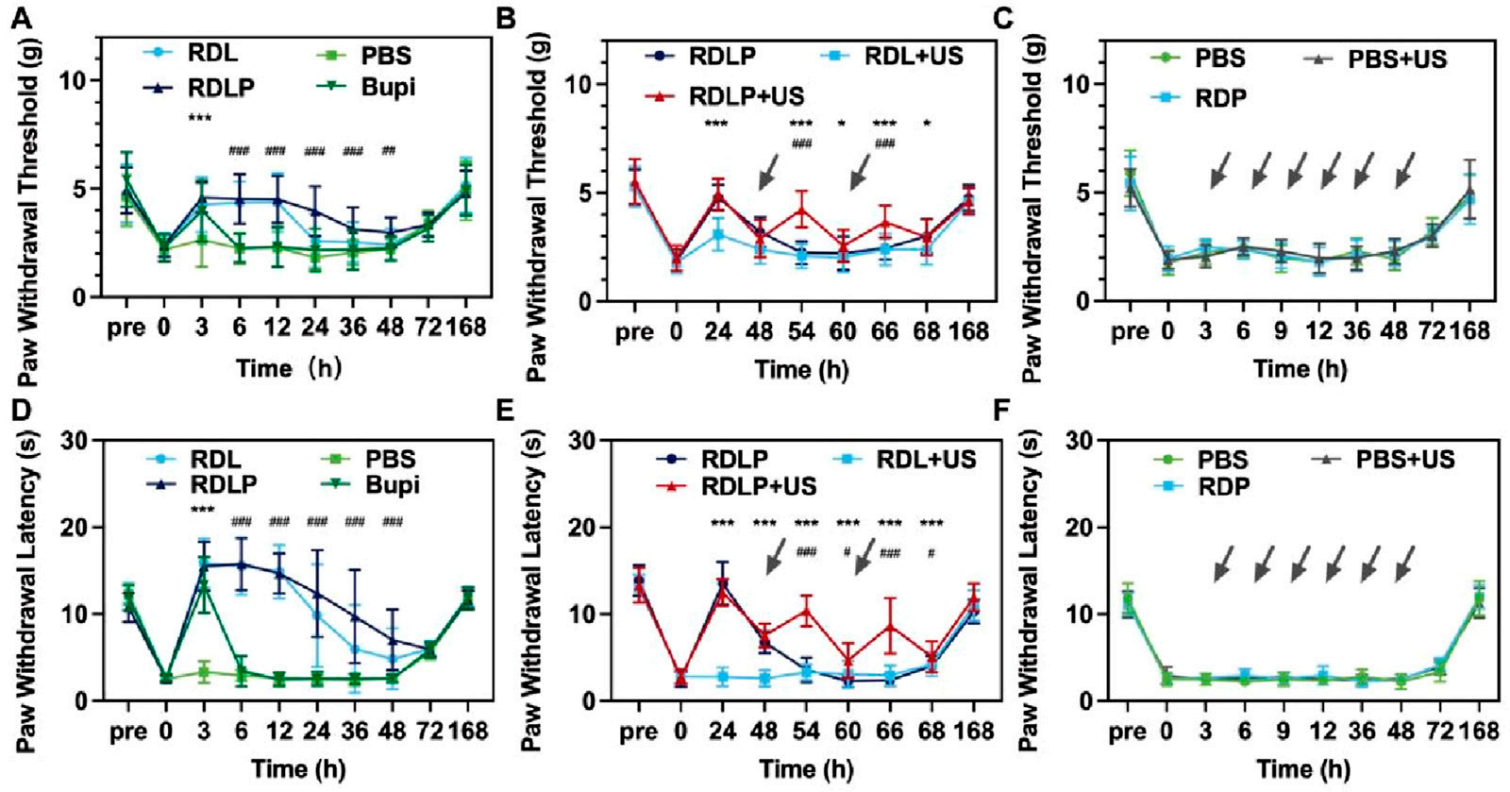
**(A)** The paw withdrawal threshold in mice exposed to various treatments is displayed (data as mean ± SD; ***p < 0.0001 for RDLP vs. levobupivacaine group, ###p < 0.0001 for RDLP vs. PBS group). **(B)** Paw withdrawal threshold results for mice under a distinct treatment approach, with dotted arrows indicating ultrasonic irradiation (data as mean ± SD; *p < 0.05 and ***p < 0.001 for RDLP + US vs. RDLP group, ###p < 0.001 for RDLP + US vs. RDLP group). **(C)** Paw withdral threshold measurements in mice treated with PBS, PBS + US, or RDP. **(D)** Thermal latency outcomes for mice across different treatments. **(E)** Thermal latency data for mice subjected to a specific strategy, featuring dotted arrows for ultrasonic irradiation. **(F)** Thermal latency results in mice administered PBS, PBS + US, or RDP (n = 5).

The study assessed the stimuli-responsive anesthetic effectiveness of RDLP nanoparticles in mouse incisional pain models. We established an incision pain model 3 h post-surgery, confirmed by reduced mechanical thresholds and thermal latency (Figure 5A) (Stratton et al., 2025). Experimental groups received perisciatic nerve injections containing levobupivacaine, DLP, or RDLP formulations. Quantitative assessment of mechanical hypersensitivity involvedbehavioral tests employing automated testing apparatus that delivered calibrated stimuli while documenting response thresholds (Figures 5A–C). Post-injection monitoring of thermal and mechanical pain occurred every 3 h for DLP, RDLP, free levobupivacaine, and PBS. PBS injection (100 μL) showed no effect on filament response (Figure 5A), whereas free levobupivacaine (100 μL) provided only a 3-h pain block. Conversely, DLP infusion (100 μL) demonstrated prolonged pain management, highlighting nanotechnology’s role in extending local anesthetic duration. However, such nanoparticles typically exhibit leakage in drug delivery (Wang et al., 2024). As previously established, decorating DLP with Rg3 based liposomes significantly reduced levobupivacaine leakage with high biocompatibility, correlating with substantially enhanced pain management efficacy.

While certain local anesthetic platforms can extend pain relief to 168 h in neuropathic pain contexts, they lack stimuli-responsive capabilities. Such nanoparticles remain unable to dynamically respond to patient health changes or clinical demands (Qiao et al., 2024). To assess ultrasound-triggered on-demand analgesia, groups receiving RDLP or DLP nanoparticles were subjected to ultrasound. Mice treated with RDLP combined with targeted ultrasound application at the sciatic nerve demonstrated markedly superior pain modulation efficacy without shortening the analgesic duration. This observation indicates ultrasound intervention effectively regulates pain recurrence following primary analgesic decline (Figure 5B), demonstrating the nanoplatform’s on-demand pain relief capability across three ultrasound cycles. This improved control stems from ultrasound-triggered acoustic droplet vaporization, enabling burst release of levobupivacaine. Conversely, DLP nanoparticles showed only moderate pain management improvement under ultrasound, likely due to *in vivo* content loss through elimination. Control groups (RDP nanoparticles alone, PBS with/without ultrasound) were established to exclude potential effects from ultrasound, liposomes, DMSN, or PFP (Figure 5C). The PBS, RDP, and ultrasound-treated PBS groups exhibited negligible analgesic effects, demonstrating the unique efficacy of ultrasound-responsive RDLP complexes in preventing postoperative incision-related pain.

Thermal nociceptive responses were quantified through behavioral observation to determine paw withdrawal latency (PWL) (Figures 5D–F). Thermal hyperalgesia resolved within 3 h, with the DLP group gradually aligning with PBS. DLP administration via the sciatic nerve extended PWL on heat plates to approximately 6 h compared to levobupivacaine, indicating potential for improved pain management (Figure 5D). RDLP provided markedly longer analgesia than DLP, attributable to Rg3 liposomes modification reduced levobupivacaine release *in vivo*. RDLP + US treatment achieved superior thermal hyperalgesia suppression, validating acoustic droplet vaporization-mediated drug release enhancement (Figure 5E). Perfluoropentane incorporation facilitated efficient gas-phase transition under sonication, optimizing ultrasound-responsive drug liberation. Minimal response alterations in PBS + US, PBS, and RDP control groups corroborated the specific therapeutic efficacy of the RDLP + US combination (Figure 5F). Complete restoration of both thermal and mechanical sensory thresholds occurred across all experimental groups by 168-h post-administration, demonstrating the effective creation and alleviation of the incisional pain model.

### 3.6 Histocompatibility assessment

**FIGURE 6:**
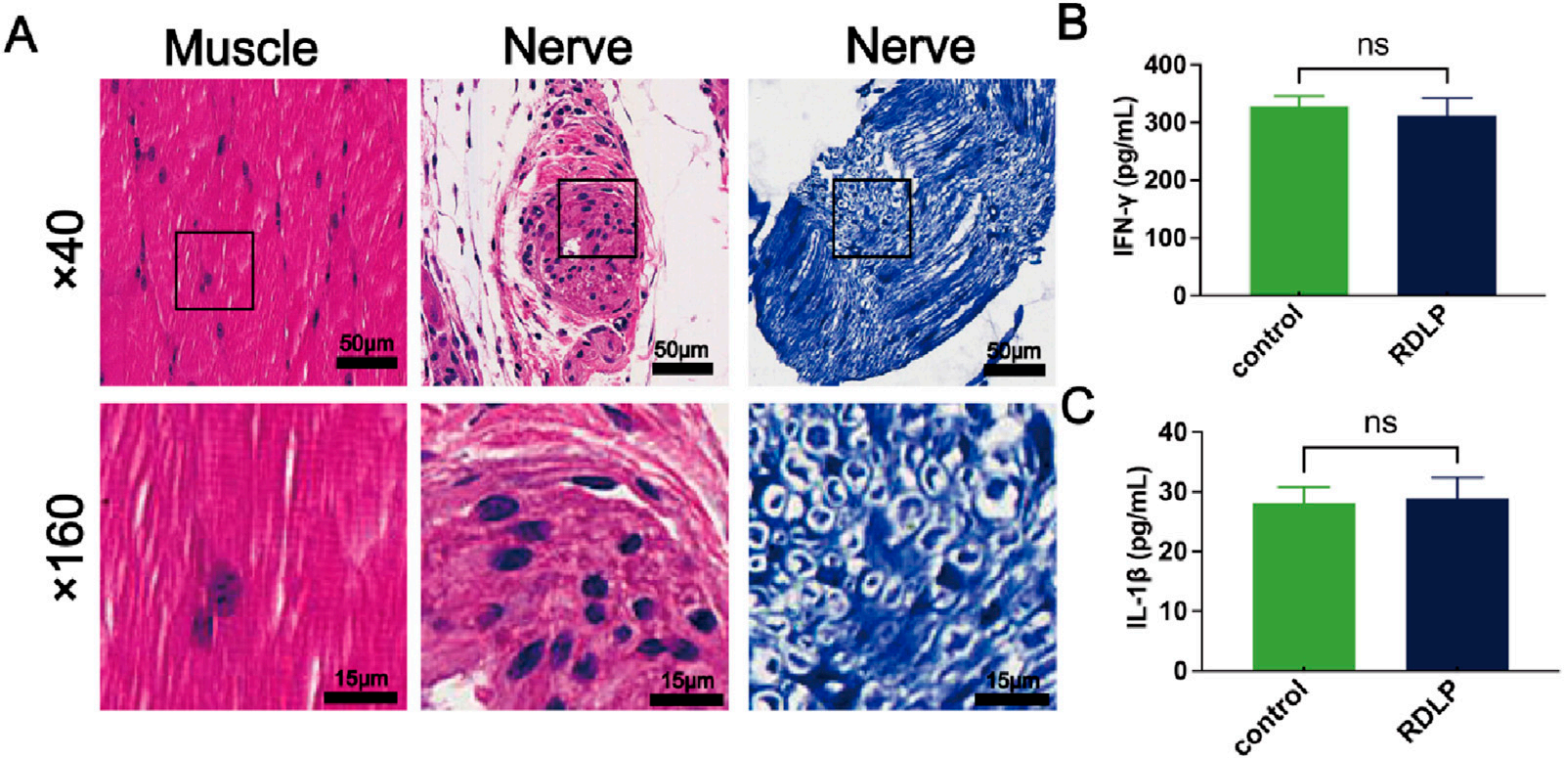
**(A)** Representative H&E (left) and toluidine blue (right)-stained images of sciatic nerve and H&E stained muscle. **(B)** IFN-γ levels of sciatic nerve and muscle after RDLP+US treatment and ultrasound treatment alone (n = 3). **(C)** IL‐1β levels of sciatic nerve and muscle after RDLP+US treatment and ultrasound treatment alone (n = 3).

For the safety assessment of RDLP in analgesia, mice receiving RDLP and ultrasound intervention were deeply anesthetized with 2% sevoflurane to ensure a painless state, followed by euthanasia via cervical dislocation at 7-day intervals after treatment. Histological sections of sciatic nerves alongside surrounding muscular and cutaneous tissues underwent hematoxylin-eosin and toluidine blue staining protocols. Histological analysis showed no evidence of swelling, tissue discoloration, or overt pathological changes in the collected specimens (Figure 6A). Considering H&E staining’s restricted resolution for neural impairment assessment, complementary toluidine blue staining was specifically performed on sciatic nerve samples. This histochemical technique selectively binds to Nissl bodies, established indicators of neuronal degeneration. Microscopic evaluation revealed only marginal peripheral nerve alterations following 7 days of ultrasound exposure, confirming that RDLP-based nanoplatforms coupled with *in vivo* ultrasound application for precision pain intervention exhibit superior tissue compatibility. Further biosafety assessments demonstrated stable cytokine profiles in perilesional tissues from RDLP-treated subjects, with no significant alterations observed in inflammatory mediator concentrations (Figures 6B,C), which demonstrated minimal toxicological impact, validating the safety profile of our proposed methodology.

## 4 Conclusion

Effective postoperative pain management requires prolonged, on-demand analgesia with minimal side effects. Here, we report a theranostic nanoplatform, RDLP, integrating ultrasound-triggered phase transition, contrast-enhanced ultrasound imaging, and intensity-tunable drug release for precision analgesia. RDLP features a core-shell design with DMSN encapsulate levobupivacaine and perfluoropentane, while Rg3-liposomes ensure biocompatibility. Ultrasound triggers PFP’s liquid-to-gas phase transition, generating microbubbles that enhance CEUS imaging for real-time drug tracking and amplify levobupivacaine release via inertial cavitation. In a mouse incision pain model, RDLP + ultrasound achieves prolonged analgesia and on-demand intensity adjustment via multiple ultrasound cycles, restoring paw withdrawal thresholds/latencies to near-baseline. Notably, RDLP shows negligible cytotoxicity and histopathological damage, ensuring safety. By leveraging ultrasound’s deep penetration and Rg3’s biocompatibility, RDLP overcomes limitations of conventional nerve blocks and light-triggered systems, offering a transformative tool for precision postoperative pain management.

## Data Availability

The original contributions presented in the study are included in the article/supplementary material, further inquiries can be directed to the corresponding authors.
